# The Peptidoglycan Pattern of *Staphylococcus carnosus* TM300—Detailed Analysis and Variations Due to Genetic and Metabolic Influences

**DOI:** 10.3390/antibiotics5040033

**Published:** 2016-09-23

**Authors:** Julia Deibert, Daniel Kühner, Mark Stahl, Elif Koeksoy, Ute Bertsche

**Affiliations:** 1 Interfaculty Institute of Microbiology and Infection Medicine Tübingen (IMIT) — Microbial Genetics, University of Tuebingen, Auf der Morgenstelle 28 E, 72076 Tuebingen, Germany; antibiotics@mdpi.com (J.D.); antibiotics@mdpi.com (D.K.); elif.koeksoy@uni-tuebingen.de (E.K.); 2 Interfaculty Institute of Microbiology and Infection Medicine Tübingen (IMIT) — Infection Biology, University of Tuebingen, Auf der Morgenstelle 28 E, 72076 Tuebingen, Germany; 3 Center for Plant Molecular Biology (ZMBP), University of Tuebingen, Auf der Morgenstelle 32, 72076 Tuebingen, Germany; mark.stahl@zmbp.uni-tuebingen.de

**Keywords:** *S. carnosus*, peptidoglycan, muropeptides, UPLC/MS, FtsEX, CcpA, carboxypeptidases

## Abstract

The Gram-positive bacterium *Staphylococcus carnosus* (*S. carnosus*) TM300 is an apathogenic staphylococcal species commonly used in meat starter cultures. As with all Gram-positive bacteria, its cytoplasmic membrane is surrounded by a thick peptidoglycan (PGN) or murein sacculus consisting of several layers of glycan strands cross-linked by peptides. In contrast to pathogenic staphylococci, mainly *Staphylococcus aureus* (*S. aureus*), the chemical composition of *S. carnosus* PGN is not well studied so far. UPLC/MS analysis of enzymatically digested *S. carnosus* TM300 PGN revealed substantial differences in its composition compared to the known pattern of *S. aureus*. While in *S. aureus* the uncross-linked stem peptide consists of a pentapeptide, in *S. carnosus*, this part of the PGN is shortened to tripeptides. Furthermore, we found the PGN composition to vary when cells were incubated under certain conditions. The collective overproduction of HlyD, FtsE and FtsX—a putative protein complex interacting with penicillin-binding protein 2 (PBP2)—caused the reappearance of classical penta stem peptides. In addition, under high sugar conditions, tetra stem peptides occur due to overflow metabolism. This indicates that *S. carnosus* TM300 cells adapt to various conditions by modification of their PGN.

## 1. Introduction

*Staphylococcus carnosus (S. carnosus*, *S. c.)* TM300 is an apathogenic, coagulase-negative, “food grade” staphylococcal species [1] commonly used as meat starter culture for raw sausages [2]. Its 2.56 Mbp genome has the highest GC content of all staphylococcal species sequenced so far. While virulence and toxicity factors are almost completely missing from the genome, most of the metabolic pathways are present. As typical for starter cultures, it harbors various sugar degradation pathways [3,4]. The general features of the peptidoglycan (PGN) of *S. carnosus* TM300 are already known. Like *Staphylococcus aureus* (*S. aureus*, *S. a.*) it belongs to the A3α-type with a penta glycine interpeptide bridge [1,5]. *N*-acetylmuramic acid (MurNAc) residues in the glycan backbone are not *O*-acetylated at position six, therefore rendering *S. carnosus* sensitive against lysozymes like all apathogenic *Staphylococcus* species [6,7].

PGN biosynthesis starts in the cytoplasm where the precursor UDP-MurNAc-pentapeptide is synthesized by the sequential addition of the stem peptide to UDP-MurNAc. At the cytoplasmic membrane, this precursor is attached to the lipid carrier undecaprenol leading to lipid I. Addition of the second sugar moiety UDP-N-acetylglucosamine (UDP-GlcNAc) results in lipid II formation, which is flipped across the cytoplasmic membrane by an enzyme, that is still highly debated in its identity, as there are two possible candidates [8]: either FtsW [9,10] or MurJ [11,12]. The *S. aureus* stem peptide consists of l-Ala-d-iGlu-l-Lys-d-Ala-d-Ala [5] with the d-iGlu being modified at the stage of lipid II to d-iGln [13,14]. The murein synthesizing enzymes—called penicillin-binding proteins (PBPs)—need at least one amidated stem peptide for their transpeptidase activity during cross-linking [15]. In general, PBPs are divided into high molecular weight (HMW) PBPs, that perform PGN synthesis, and low molecular weight (LMW) PBPs, which are PGN hydrolases [16]. *S. aureus* contains four native synthesizing PBPs and homologues of all of these can be found in the genome of *S. carnosus* TM300. PBP2 is considered to be the main enzyme for cell wall biosynthesis in staphylococci and is localized to the division septum by binding to its transpeptidation substrate lipid II [17]. PBP2 is the only bifunctional PBP in *S. aureus* and *S*. *carnosus*. It catalyzes the polymerization of the glycan moieties of lipid II by its transglycosylase activity resulting in glycan chains of alternating β-1,4-linked GlcNAc-MurNAc residues. In addition PBP2 is able to cross-link adjacent stem peptides by its transpeptidase activity [18,19]. Transpeptidation occurs between the d-Ala on position four of the donor stem peptide and the N-terminal Gly of the interpeptide bridge of the acceptor stem peptide by the expense of the last d-Ala of the donor stem peptide. The resulting tetra stem peptide can serve as an acceptor for further cross-linking reactions (Supplementary Materials Figure S1). The penta stem peptide of the first acceptor or of free acceptors in *S. aureus* however has never been reported to be modified in any way.

In *S. aureus* there are three additional monofunctional PBPs: (1) the essential enzyme PBP1, which is crucial for the cell division mechanism [20]; (2) the non-essential PBP3, which is involved in autolysis [21] and (3) the non-essential PBP4, which belongs to the low molecular weight PBPs and normally is considered to be a murein hydrolase. In *S. aureus* however, PBP4 is responsible for the high degree of cross-linking [22] and for the resistance against β-lactams in community-acquired methicillin-resistant *S. aureus* strains (caMRSA) [23]. There are two possible candidates for PBP4 in *S. carnosus* TM300 found in KEGG [24]: SCA_0291 with 64.7% or SCA_2445 with 37.6% sequence identity to *S. aureus* COL, respectively. While PBPs are generally considered to contain a non-cleavable signal peptide functioning as a single N-terminal transmembrane anchor [16], these three proteins are all predicted to contain a second transmembrane domain on the C-terminus [25].

The concerted actions of transglycosylase and transpeptidase reactions of the PBPs build a mesh-like macromolecule that surrounds the whole cell. During cell division this PGN sacculus has to be divided into two parts without compromising integrity. *S. aureus* is a spherical bacterium that divides along three orthogonal planes over the course of three division cycles [26]. While it was long believed that spheres like staphylococci grow by PGN synthesis at the cell division site only, recent investigations by super-resolution microscopy revealed additional peripheral PGN biosynthesis catalyzed by PBP4 [27]. Cell division in all bacteria depends on formation of the FtsZ-ring and the sequential recruitment of various proteins such as FtsE and FtsX to the divisome [28,29]. FtsEX is similar to an ABC transporter on a structural and sequence level [30] with FtsE being the ATP binding subunit and FtsX being the integral membrane part. Rather than being transporters, these two proteins are proposed to regulate the activity of murein hydrolases like PcsB in *Streptococcus pneumoniae* [31,32,33] and the amidases AmiA and AmiB—both via EnvC—in *E. coli* [34]. In *Bacillus subtilis*, FtsX interacts with the endopeptidase CwlO [35]. Genes encoding for FtsE and FtsX can also be found in *S. carnosus* TM300. However, the resulting proteins do not possess sequence similarity but only structural similarity. The role of FtsE and FtsX during synthesis of the PGN sacculus has not been investigated in staphylococci so far.

The PGN sacculus can be isolated and digested by a muraminidase such as mutanolysin into fragments called muropeptides. Muropeptides are disaccharide units with peptide moieties that can be cross-linked to other stem peptides and disaccharides thereby resulting in oligomeric structures [36]. During the analyses of *S. carnosus* TM300 muropeptides, we observed degradation of the first acceptor stem peptides from five (pentapeptide) to three (tripeptide) amino acids, which is in contrast to the known muropeptide pattern of *S. aureus* [37,38]. This gave us reason to perform a detailed Ultra Performance Liquid Chromatography (UPLC) and UPLC mass spectrometry (UPLC/MS) analysis of the *S. carnosus* TM300 muropeptide pattern. Our results show that almost all stem peptides that were not part of a cross-link contain only three amino acids (l-Ala-d-iGln-l-Lys) under standard conditions. However, overexpression of HlyD-FtsE-FtsX, a putative protein complex interacting with PBP2, partly inhibited this degradation process. A similar effect was observed for high glucose and fructose concentrations.

## 2. Results

### 2.1. Peptidoglycan Analysis

**Figure 1:**
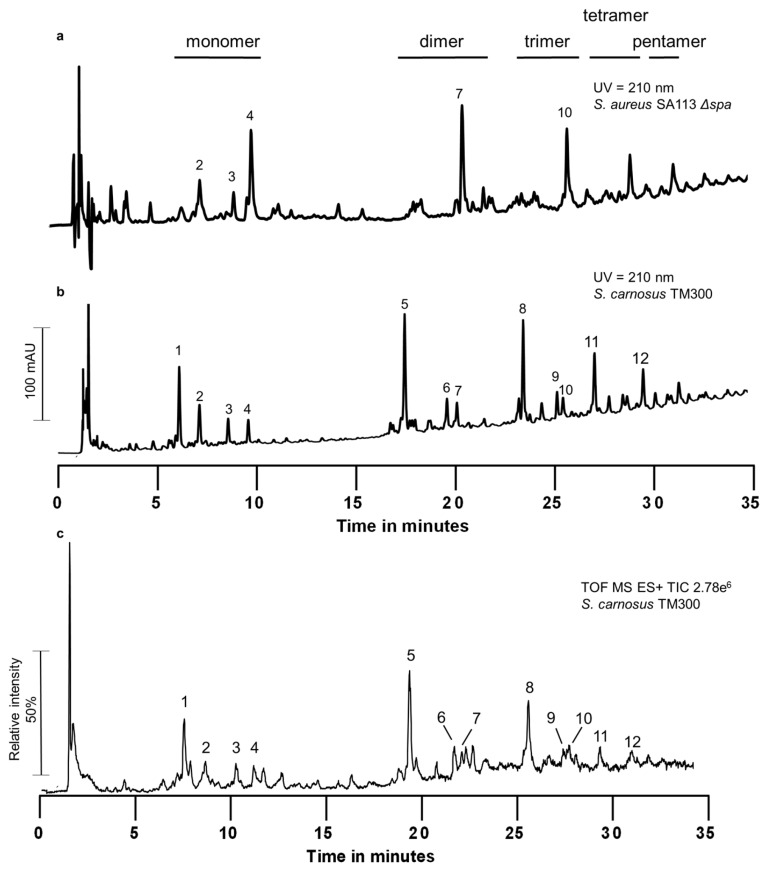
Muropeptide profile of *S. carnosus* TM300 by UPLC and UPLC/MS. Peptidoglycan was isolated, digested into muropeptides and analyzed by reversed phase UPLC and UPLC/MS. (**a**) Muropeptide profile of *S. aureus* SA113 Δ*spa* obtained by UPLC. Muropeptide peaks that are identical with the ones of *S. carnosus* TM300 are numbered; (**b**) Muropeptide profile of *S. carnosus* TM300 as obtained by UPLC; (**c**) TIC of UPLC/MS analysis of *S. carnosus* TM300 peptidoglycan. Muropeptides of *S. carnosus* TM300 exhibit shorter retention times compared to *S. aureus* SA113 Δ*spa*. UPLC/MS analysis revealed the presence of stem peptides containing only three amino acids instead of five. Masses of indicated peaks are shown in Table 1 including molecule composition and proposed molecular formula. Retention time of peaks in the UPLC (quaternary pumping system) was about 1 min longer than in the binary UPLC/MS system. Therefore, in Table 1 only TIC retention times are given (TIC: Total Ion current, recorded in positive mode).

We analyzed the PGN of *S. carnosus* TM300 by UPLC (Figure 1) and observed that it differed substantially from the known pattern of *S. aureus* [37,38]. The whole muropeptide pattern of *S. carnosus* was shifted to shorter retention times compared to *S. aureus*. We performed UPLC-MS analysis to determine the chemical composition of the muropeptide peaks (Table 1). All main peaks in the monomeric as well as in the cross-linked fractions (dimer to pentamer fraction) contained muropeptides with stem peptides only consisting of three amino acids: l-Ala-d-iGln-l-Lys, explaining the shift in retention time. In *S. aureus*, the non-cross-linked stem peptides of monomeric muropeptides as well as the acceptor stem peptide of multimeric muropeptides harbor the complete penta stem peptide. These penta muropeptides are also present in *S. carnosus*, but in small amounts compared to the respective tri muropeptide. The cross-linked stem peptides are still tetra peptides and do not get degraded into tri peptides, leading to Tri-Tetra_n_ muropeptides (Table 1). During the course of our experiments, we realized that the amount of different muropeptides varied and was influenced by genetic factors as well as composition of the medium.

### 2.2. Search for New Proteins Involved in PGN Biosynthesis

We performed a Bacterial-Two-Hybrid (BTH) screen using a genomic library of *S. carnosus* TM300 to search for new interaction partners of PBP2. This bifunctional transglycosylase/transpeptidase is considered to be one of the main players of PGN biosynthesis in *S. aureus* [16,17,18,19,39,40]. Among others, we found the so far uncharacterized protein SCA_1997, a conserved hypothetical protein that belongs to the HlyD family of secretion proteins (K02005). Therefore, we refer to this gene as *hlyD*. Bioinformatical analyses using the Kyoto Encyclopedia of Genes and Genomes (KEGG) [24] showed that if *hlyD* is present in *Bacillus* *spec.* and *Staphylococcus* *spec.* it is always located in an operon with two other genes. Each of them possesses its own start codon but they share one Shine-Dalgarno sequence and one promotor region. The first gene (SCA_1996) encodes for a putative ABC transport system ATP-binding protein (K02003) with structural similarity to FtsE of *E. coli*. The second gene (SCA_1995) is predicted to produce an ABC transport system permease protein (K02004) with structural similarity to *E. coli* FtsX [30,41]. So far, there is no function described for the staphylococcal HlyD, FtsE and FtsX proteins.

**Figure 2:**
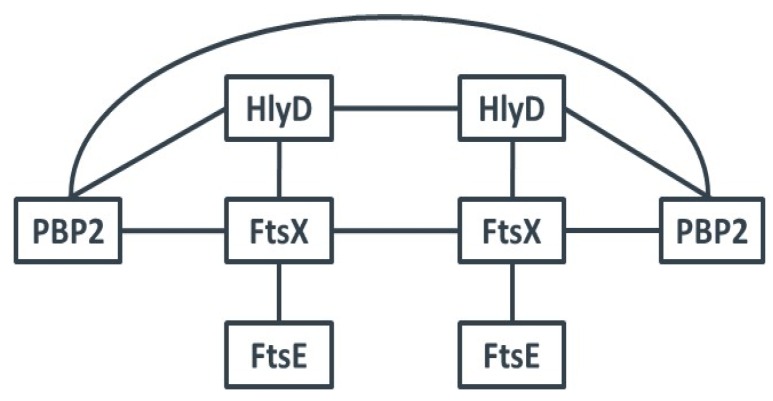
Protein-Protein interactions. BTH analyses revealed that PBP2 interacted with itself as well as with HlyD and FtsX. HlyD also interacted with FtsX. In addition, FtsX interacted with itself and with FtsE. There was no self-interaction of FtsE determined, and FtsE did not interact with HlyD or PBP2.

We cloned the genes of each of these proteins into the two vectors of the BTH system and performed interaction studies with all possible pairs (Table 2). PBP2 interacted with itself as well as with HlyD and FtsX. HlyD also interacted with FtsX. In addition, FtsX interacted with itself and with FtsE. There was no self-interaction of FtsE detected, and FtsE did not directly interact with HlyD (Figure 2). The interactions of the full length proteins confirmed our genetic screen results, and suggest that HlyD, FtsX and FtsE are involved in the same or in closely related biosynthetic pathways, most likely in cell wall biosynthesis and/or cell division.

**Figure 3:**
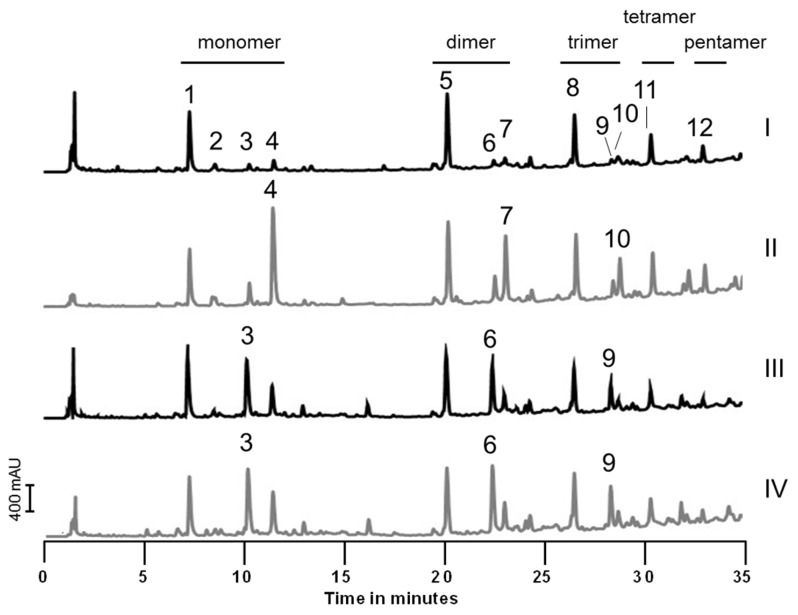
Variations in muropeptide profile caused by genetic and metabolic factors. PGN from different *S. carnosus* TM300 variants was isolated, digested into muropeptides and analyzed by UPLC. (**I**) *S. carnosus* TM300 grown in the presence of 25 mM xylose; (**II**) *S. carnosus* TM300 pPTX-*hlyD-ftsEX* induced with 25 mM xylose; (**III**) *S. carnosus* TM300 pPTX-*hlyD-ftsEX* repressed with 25 mM glucose; (**IV**) *S. carnosus* TM300 grown in the presence of 25 mM glucose. Overexpression of *hlyD-ftsEX* led to an accumulation of penta muropeptides (peak 4, 7 and 10 in panel **II**) which was not observed when the wild type strain was grown in the presence of 25 mM xylose. Addition of 25 mM glucose in the medium lead to an increase in tetra muropeptides independent of the plasmid pPTX-*hlyD-ftsEX* (peaks 3, 6 and 9 in panel **III** and **IV**, respectively).

Therefore, we deleted the whole operon and examined the resulting mutant strain *S. carnosus* TM300 Δ*hlyD-ftsEX*. We did not observe any difference in growth behavior under standard BM conditions (Supplementary Materials Figure S2A), in minimal M9 medium (Supplementary Materials Figure S2B), or when the amount of NaCl in the medium was varied (Figure S2C). In addition, deletion of this operon did not have any effect on the muropeptide composition (Figure S3A). However, when the three genes were overexpressed from a plasmid harboring a xylose inducible promoter, all peaks with muropeptides containing a penta stem peptide were highly elevated while the tri muropeptides were still present in comparable amounts. Muropeptides with tetra stem peptides were slightly elevated as well. As 25 mM xylose itself had no effect on the wild type strain, the observed effect is due to overexpression of the *hlyD-ftsEX* operon (Figure 3) while overexpression of *hlyD* or *ftsEX* had no effect on muropeptide composition (Supplementary Materials Figure S3B). Astonishingly, we also observed elevated tetra and—to a lower extent—penta muropeptides when we repressed plasmid gene expression with 25 mM glucose, indicating that the PGN of *S. carnosus* is influenced by genetic as well as metabolic factors.

### 2.3. Precursor Analysis

**Figure 4:**
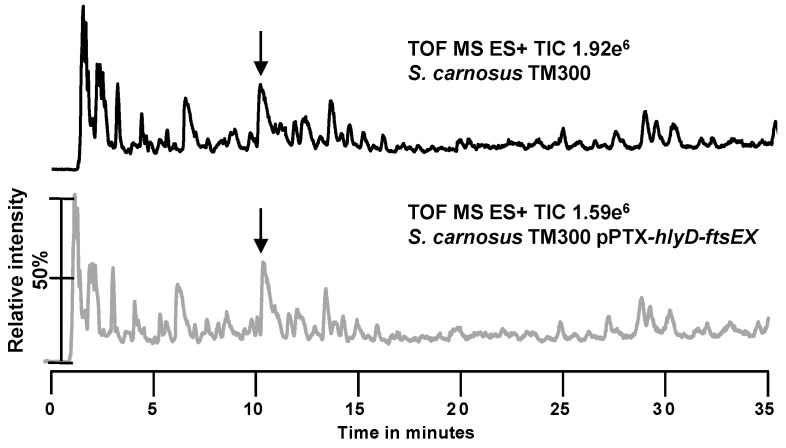
UPLC/MS analysis of the PGN precursor. *S. carnosus* TM300 wild type and *S. c.* TM300 pPTX-*hlyD-ftsEX* were grown in B medium supplemented with 25 mM xylose. Precursors were concentrated, isolated and analyzed by UPLC/MS. Peaks highlighted by arrows were identified to harbor the same masses including the mass for the classical precursor UDP-MurNAc-pentapeptide (1149.37 g/mol). Masses for UDP-MurNAc-tripeptide (1007.2774 g/mol) or UDP-MurNAc-tetrapeptide (1078.3145 g/mol) were not detected in any of the analyses.

To examine if *S. carnosus* TM300 synthesizes already shortened variants of the classical PGN precursor UDP-MurNAc-pentapeptide, we accumulated and analyzed the PGN precursor of the wild type strain *S. carnosus* TM300 and of the overexpression strain *S. carnosus* TM300 pPTX-*hlyD*-*ftsEX* (Figure 4). In *S. aureus*, the PGN precursor is the UDP-MurNAc-pentapeptide with a mass of 1149.37 g/mol. In both tested *S. carnosus* strains, this mass was only found in a broad peak group at about 11 min in the TIC of the UPLC-MS. None of the other masses obtained correlated with the UDP-MurNAc-tripeptide (1007.2774 g/mol) or UDP-MurNAc-tetrapeptide (1078.3145 g/mol). This shows that *S. carnosus* TM300 only synthesizes the classical precursor UDP-MurNAc-pentapeptide and truncation to the tetra and tri stem peptide must occur at a later time point.

### 2.4. Investigation of a Putative l,d-Carboxypeptidase

A logical explanation for the observed truncation of the penta stem peptide to a tri stem peptide would be an l,d-carboxypeptidase (ld-CP) activity. Even though such an activity has never been described for staphylococci, we found one potential candidate in the genome of *S. carnosus* TM300: SCA_0214 is annotated as a cytoplasmic muramoyl-tetrapeptide carboxypeptidase that hydrolyses the bond between a di-basic amino acid and the C-terminal d-alanine in the tetra peptide moiety of PGN. An orthologous gene was not found in *S. aureus* and no paralog was annotated in *S. carnosus* TM300. Therefore, a deletion mutant of SCA_0214 was created in *S. carnosus* TM300. However, deletion of this gene did not alter the PGN pattern compared to the wild type strain (Supplementary Materials Figure S4A). When we overexpressed this gene, we found the resulting protein to accumulate in the cytoplasm and not be transported to the supernatant (Supplementary Materials Figure S4B). As truncation of the stem peptide most likely occurs outside the cell, we exclude SCA_0214 as the sought-after ld-CP.

### 2.5. Influence of Sugars on PGN Composition

**Figure 5:**
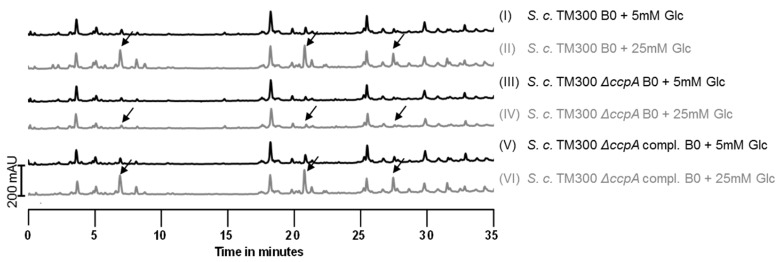
Deletion of *ccpA* prevents the effect of high sugar concentrations. The catabolite repressor gene *ccpA* was deleted in *S. carnosus* TM300. PGN of the wild type, the mutant and the complemented strain grown with 5 or 25 mM glucose was isolated and analyzed by UPLC. In the *ccpA* deletion mutant, 25 mM glucose did not cause accumulation of tetra muropeptides (arrows in panel 4), while complementation with a constitutively expressed *ccpA* gene completely restored this effect (panel 6).

As we had observed that high concentrations of glucose (25 mM) led to an increase in muropeptides with tetra stem peptides, we tested other C-sources in this concentration as well. While fructose had the same effect, the muropeptide pattern in the presence of glycerol or ribose was unchanged. PGN of cells grown in B0 medium (B medium without an additional C-source) was also unaffected (Supplementary Materials Figure S5A). Therefore, we examined whether this observation was due to overflow metabolism and deleted the gene for the catabolite control protein A (*ccpA*). CcpA mainly functions as a gene repressor, but it is also involved in the transcription activation of genes involved in fermentation and overflow metabolism [42,43]. It is itself activated by glucose intermediates like glucose-6-phosphate and fructose-1,6-bisphosphate [44]. Indeed, the effect of high amounts of glucose on PGN composition was lost in the Δ*ccpA* mutant (Figure 5). In the complemented mutant, the *ccpA* gene was constitutively expressed from a plasmid, and addition of 25 mM glucose to the medium again caused the appearance of muropeptides with tetra stem peptides in addition to tri stem peptides. Alterations in the PGN pattern of *S. carnosus* TM300 were not solely reduced to high glucose concentrations and were lost in a *ccpA* deletion mutant. Therefore, this effect can be explained by an overflow metabolism when an abundance of nutrients is present in the medium rather than solely by a glucose effect. In *S. aureus*, SA113 an increased glucose concentration showed no effect on the muropeptide pattern (Supplementary Materials Figure S6).

### 2.6. Deletion of ccpA Causes a Decrease in Colony Size and Growth Rate

**Figure 6:**
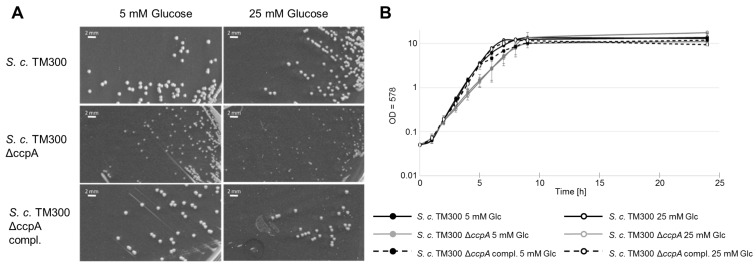
The Δ*ccpA* mutant showed a decrease in colony size and a reduction in growth rate. (**A**) On solid media colonies, the deletion mutant appeared clearly smaller than the parental strains. The glucose concentration seemed to have only a minor influence on colony size; (**B**) In liquid media, *S. carnosus*TM300 Δ*ccpA* also showed a growth defect compared to the wild type strain. In both cases, the effect could be complemented. The influence of the glucose concentration in both experiments was negligible. (Bar size: 2 mm; Glc: glucose).

While the observed variation in PGN composition was lost in the *ccpA* deletion mutant, this strain was influenced in its growth behavior. There was a decrease in colony size (Figure 6 and Table 3) of the *S. carnosus* TM300 Δ*ccpA* mutant of ~60% compared to the wild type strain. Complementation restored the mutant phenotype completely. In liquid culture, the *S. carnosus* TM300 Δ*ccpA* mutant showed a growth defect during log phase compared to the wild type strain that was restored again by complementation (Figure 6). Growth rate of the mutant was reduced to 60% in low glucose concentration and to 68% in high glucose concentrations compared to the wild type. This effect was restored by complementation (Table 4). Therefore, in these experiments, the glucose concentration itself had only minor influence, indicating observed effects on the PGN are specific.

## 3. Discussion

### 3.1. The Peptidoglycan Composition of S. carnosus TM300

UPLC/MS analysis of the PGN of *S. carnosus* TM300 revealed a muropeptide pattern different from the one known of *S. aureus*. Instead of stem peptides containing the classical five amino acids l-Ala-d-iGln-l-Lys-d-Ala-d-Ala) typical for *S. aureus*, most stem peptides were degraded to tri peptides missing the two d-Ala. This is the case for monomeric muropeptides (Figure 1a, peak 1) as well as for all cross-linked muropeptides, in which the acceptor stem peptide was also degraded, while the former donor stem peptides are still classical tetra peptides (Figure 1a, peaks 5, 8, 11 and 12). We were able to identify the cross-linked muropeptides up to the pentamer (Tri-Tetra_4_). Acceptor stem peptides with penta peptides were also found (Figure 1a, peaks 4, 7 and 10), but in small amounts only. We also found tetra muropeptides containing five (Figure 1a, peak 3) or six to nine glycine residues in the interpeptide bridge (Figure 1a, peak 2). The latter indicates that these muropeptides had been released again from mature PGN. This peak was not influenced by the genetic and metabolic factors tested.

In *S. aureus* PGN—the best studied PGN in staphylococci—this degradation was never observed [37,38,45]. Tri stem peptides are a classical feature of *E. coli* [36] but also of other firmicutes like *Bacillus subtilis*, where the PGN is modified after cross-linking has occurred. Interestingly, we observed the length of the *S. carnosus* TM300 stem peptide not only to be influenced by genetic factors (in our study *hlyD-ftsE-ftsX*) but also by the composition of the growth medium (see below). Especially 25 mM glucose, a concentration frequently used for plasmid repression, caused an increase in muropeptides with tetra stem peptides. Changes in stem peptide composition due to medium ingredients have been reported for example for *Caulobacter crescentus*. It incorporates glycine at position five, even if only traces of free glycine are present in the growth medium [46]. When glycine is depleted in the medium, *S. aureus* PGN is directly cross-linked and lacks the interpeptide bridge [47]. Cells then enter the stationary phase, indicating this to be a protection mechanism.

### 3.2. The hlyD-ftsE-ftsX Operon

A gene for HlyD (hemolysin D) is present in e.g., *Escherichia coli* (*E. coli*) but the orthologue could not be found in staphylococci. The database KEGG and a Blast search for orthologous genes did not lead to any positive results. However, SCA_1997, which was identified in the BTH screen with a genomic library of *S. carnosus* TM300 as a putative interaction partner for PBP2, was annotated as a conserved hypothetical protein belonging to the HlyD family of secretion proteins. The nomenclature was maintained as SCA_1997 and orthologous genes possess a short HlyD motif that is eponymous for these proteins.

Like the *E. coli* HlyD, the staphylococcal HlyD is highly similar to the membrane fusion component of the RND family of transporters (RND: Resistance, Nodulation, Cell Division). As the proposed function is to facilitate and enable transport (e.g., resistance through efflux) by bringing the inner and outer membrane together, most members of this family are found in Gram-negative bacteria [48]. Nevertheless, also in Gram-positive bacteria, there are a few representatives of this family. But *hlyD* is not omnipresent in *Staphylococcus*. Only about 47% (34 out of 73) of all annotated staphylococci harbor a copy of *hlyD*. In contrast, in *Bacillus*, 98% of strains (112 out of 114 strains sequenced) possess this gene. There is also no correlation between *hlyD* and pathogenicity, as it can be found throughout the whole genus of staphylococci.

While the *S.* *carnosus* TM300 FtsEX complex has no sequence similarity to FtsEX of *E. coli*, the predicted structures of the respective proteins are very similar. An *ftsEX* depletion mutant in *E. coli* forms filaments, and growth in LB medium was shown to be salt dependent as the septal ring does not properly assemble in the absence of FtsEX and salt [30]. However, growth of the *S. carnosus* TM300 *hlyD-ftsEX* mutant was not affected by the absence or presence of NaCl nor by the use of minimal medium M9 (Supplementary Materials Figure S2) and we did not observe any obvious changes in cell appearance. This indicates the septal ring in staphylococci does not necessarily need *ftsEX* for stabilization.

Our BTH results are in accordance with known results for PBP2 of *S. aureus* which was also shown to self-interact [49]. In our experiments, FtsX also interacted with HlyD, with itself and with FtsE (Figure 2 and Table 2). We could not detect homodimerization of FtsE as was published for the *Streptococcus pneumoniae* orthologue. In this organism, both FtsE and FtsX formed homodimers and interacted with each other [31]. In various species, FtsE and FtsX have been shown to interact with each other and thereby activate murein hydrolases. This includes PcsB of *Streptocccus pneumoniae* [31,32,33], the endopeptidase CwlO of *Bacillus subtilis* [35] and an amidase of *E. coli* that was activated by FtsEX via EnvC [34]. Our results from *S. carnosus* point to a role for HlyD-FtsEX in the regulation of a carboxypeptidase, as the overexpression of the *hlyD-ftsEX* operon led to an increase in muropeptides containing penta stem peptides. To a minor extent, tetra stem peptides were also observed (Figure 3). Penta stem peptides of *S. carnosus* are normally degraded into tri stem peptides by a so far unknown enzyme(s). We propose that HlyD-FtsEX must have an inhibitory effect on this/these enzyme/s, as deletion of the whole operon did not alter the muropeptide pattern and therefore did not influence degradation activity. In addition, only overproduction of all three proteins caused an increase in penta and tetra muropeptides, indicating a necessity for complex formation for activity. As tri muropeptides are present in similar amounts to pentas, inhibition of the degradation enzymes is not complete and other factors might contribute to their regulation. This is corroborated by the notion that the *hlyD-ftsEX* operon is not essential and its deletion had no effect on bacterial growth under various conditions tested.

### 3.3. Investigation of a Putative l,d-Carboxypeptidase (SCA_0214)

The PGN of *S. carnosus* TM300 mainly consists of tri and tetra peptide structures (Table 1) suggesting l,d-carboxypeptidase (ld-CP) activity. Bioinformatical analysis led to only one possible candidate in *S. carnosus* TM300: SCA_0214, which is located between SCA_0213 (an NAD dependent epimerase/dehydratase family protein) and SCA_0215 (*ilvE*, a branched chain amino acid aminotransferase). In contrast to *S. carnosus* TM300, a gene for an ld-CP is missing from the genome of *S. aureus*. For instance, the genome of *S. a.* USA300 does not contain a homologous gene for SCA_0214. Instead, the two genes SCA_0213 and SCA_0215 are located directly next to each other. The absence of a putative ld-CP is in accordance with the presence of only penta and tetra stem peptides in *S. aureus* PGN [37]. However, deletion of SCA_0214 in *S. carnosus* TM300 had no effect at all on the muropeptide pattern. It still harbored mainly tri stem peptides (Figure S4A). Even if SCA_0214 was only able to cleave off d-Ala from a tetrapeptide but not from a pentapeptide, as could be expected from a carboxypeptidase, its deletion should have resulted in an accumulation of tetrapeptides. As this is not the case, we exclude SCA_0214 as being responsible for the occurrence of tripeptides in *S. carnosus* TM300.

But what could be the role of SCA_0214? Overproduction resulted in protein accumulation in the cytoplasm. This is in accordance with the ld-CP sequence analysis that revealed neither a TAT (Twin Arginine Translocation) nor a Sec (Secretion) sequence. This could point to a role in the cytoplasm, but could also be a mere side effect of protein overproduction.

But which enzyme(s) could cause the reduction from penta to tri stem peptides? As trimming of the stem-peptides during PGN maturation is widely seen in eubacteria, *S. carnosus* might be “normal”, while actually *S. aureus* is an exception by keeping its pentapeptide intact. Another bioinformatical search in KEGG revealed three annotated dd-CPs in *S. carnosus*: (1) SCA_0291, the homologue of *S. aureus* PBP4, which is actually a PGN synthase responsible for secondary cross-linking of PGN [22]; (2) SCA_2445, which is also assigned as an *S. aureus* PBP4 homologue, but with less homology; and (3) SCA_1643 that is orthologous to LdcB of *Streptococcus pneumoniae* and *B. subtilis* but has no orthologue in *S. aureus*. Both enzymes had originally been annotated as d,d-CP (*dacA*) but were recently shown to degrade tetra peptides from isolated PGN into tri peptides in vitro and were therefore renamed [50]. SCA_2445 could be the enzyme that creates tetra peptides, and tri peptides are then produced by SCA_1643. We tried to construct deletion mutants of SCA_1643 and SCA_2445 but were unsuccessful.

### 3.4. Influence of Sugars on the Muropeptide Composition

As we had observed an altered PGN pattern when we repressed plasmid gene expression with 25 mM glucose, we tested the influence of various carbon sources on the PGN pattern of *S. carnosus* TM300. We found an increase of tetra muropeptides when the cells of *S. carnosus* TM300 were grown with either 25 mM glucose (Figure 5, chromatogram 2) or fructose (Figure S5A). Again, tri muropeptides were still present in comparable amounts to these tetra muropeptides. No increase in tetra muropeptides was observed when supplemented with 25 mM xylose (Figure S5A), the standard gene induction conditions for plasmid pPTX [51,52] that served as a control.

In Gram-positive bacteria, the catabolite control protein A (CcpA), a global regulator of carbon source metabolism, regulates cellular processes that are influenced by the presence of glucose [53]. Therefore, we deleted the *ccpA* gene and analyzed the PGN composition again under low and high glucose concentrations. As expected, no elevated tetra peaks were observed in the *ccpA* deletion mutant under high glucose conditions. Instead, the muropeptide pattern was identical to the one obtained from cells grown in 5 mM glucose (Figure 5, chromatograms 3 and 4). Complementation with a plasmid constitutively expressing *ccpA* restored the original phenotype, showing that the appearance of tetra muropeptides can be traced back to the influence of glucose and fructose intermediate products on CcpA. Moreover, deletion of *ccpA* also affected cell growth. The deletion strain show a reduced growth rate in exponential phase and a severe decrease in colony size compared to the wild type strain, indicating a major role for CcpA in the metabolism of *S. carnosus* TM300.

Glucose and fructose are both metabolized to fructose-1,6-bisphosphate (FBP), an intermediate product of glycolysis. As alterations on the PGN pattern of *S. carnosus* TM300 did not only occur under high glucose concentrations, this effect could be rather explained by a general overflow metabolism instead of a glucose effect. However, glucose not only serves as a nutrient but also the expression of metabolic genes [54,55] and virulence factors [56] are influenced by glucose.

But how can glucose concentration influence the PGN composition? The increase in tetra muropeptides speaks again for a partial inhibition of a so far unknown ld-CP, although an increase in penta peptide structures was also detected. Taken together, this points to an influence of *ccpA* and *hlyD-ftsEX* on the stem peptide degrading enzyme(s) which is albeit not a complete inhibitory effect as tri stem peptides are also still present.

So far we do not understand why *S. carnosus* TM300 trims its stem peptides in a way that is also seen for other bacteria, but *S. aureus* does not. It was just shown that in *E. coli* the eight dd-CPs are active under different conditions. For example, PBP5 (*dacA*) is mainly active at neutral pH, while PBP6b is only expressed and active at acidic pH. Loss of either enzyme results in morphological defects when the cells are incubated at the respective pH. We, however, did not observe any obvious changes in the morphology of our strains during overexpression of the *hlyD*-*ftsE*-*ftsX* operon or under high glucose conditions. But the notion that even under these conditions tripeptides are present in similar amounts as the newly appearing penta or tetrapeptides can be interpreted as a hint for an important role of the tri peptides in the PGN of *S. carnosus* TM300.

## 4. Materials and Methods

For *strains*, *plasmids* and *oligonucleotides* used please refer to Supplementary Materials Table S1: Bacterial strains, Table S2: Plasmids and Table S3: Oligonucleotides.

### 4.1. Media

Basic medium (B medium) for *Staphylococci* consisted of Soy Peptone (10 g; Plato, Koblenz, Germany), Yeast Extract (5 g; Deutsche Hefewerke, Nuernberg, Germany), NaCl (5 g; Carl-Roth, Karlsruhe, Germany), Glucose (1 g; Carl Roth) and K_2_HPO_4_ (1 g; Applichem, Darmstadt, Germany). Deionized water was added to a final volume of 1 liter and pH was adjusted to 7.2.

LB medium for *E. coli* consisted of Peptone (10 g; Plato), Yeast Extract (5 g; Deutsche Hefewerke) and NaCl (5 g; Carl Roth). Deionized water was added to a final volume of 1 liter and pH was adjusted to 7.2. MacConkey agar was prepared according to the manufactures’ instructions (Carl Roth). Minimal media M9 for staphylococci consisted of Na_2_HPO_4_ (6 g; Carl Roth), KH_2_PO_4_ (3 g; Fisher Chemicals), NH_4_Cl (1 g; Merck Darmstadt, Germany) and NaCl (0.5 g; Carl Roth). Deionized water was added to a final volume of 1 L, the media was autoclaved and supplemented with thiamine (2 µg/mL; Sigma Aldrich, Munich, Germany), MgSO_4_ (1 mM; Carl Roth) and casamino acids (0.2%; BD Bioscience, Heidelberg, Germany). Cells were routinely grown at 37 °C either in liquid culture in baffled Erlenmeyer flasks (1:5 ratio) with shaking or on 1.5% agar plates.

### 4.2. Reagents

Unless otherwise stated, all reagents were bought from Sigma-Aldrich.

### 4.3. Plasmid Construction for Deletion Mutants and Overexpression

Chromosomal DNA from *S. carnosus* and *S. aureus* was isolated [57] using lysostaphin (0.5 mg/mL) for cell lysis. SCA_1995-1997(*ftsX-ftsE-hlyD*): For deletion of the operon SCA_1995-1997 ,the pBT2 [58] knockout vector was used. For overexpression of HlyD, FtsE and FtsX, the genes SCA_1997, SCA_1995-1996 and SCA_1995-1997 were cloned into the xylose inducible plasmid pPTX [52] and cut with the same enzymes. SCA_0214 (putative ld-CP): For deletion, the knockout vector pGS1 was used [59]. The overexpression plasmid was constructed in the xylose inducible plasmid pPTX [52] and cut with the same enzymes. SCA_1342 (*ccpA*): For deletion, knockout vector pGS1was used [59]. For constitutive complementation, SCA_1342 was ligated into pRAB11-EF-TU, cut with the same enzymes. SAOUHSC01850 (*ccpA*) was deleted from *S. aureus* SA113 Δ*spa* using the pMAD knockout vector [60] and Gibson assembly [61] for cloning. For overexpression, pRAB11-EF-TU-SAOUHSC01850 was electroporated first into RN4220 and then into *S. aureus* SA113 Δ*spa* [62]. The correctness of all plasmids was confirmed by sequencing before transformation. *S. carnosus* TM300 and *S. aaureus* SA113 Δ*spa* were transformed by electroporation [62]; for *E. coli*, transformation chemocompetent cells were used [63].

### 4.4. Gene Deletion

All knockout procedures were performed according to the respective protocols of the vectors. While the pMAD system does not imply the usage of a resistance cassette, the other two knockout vectors were constructed with an *ermB* (erythromycin B) resistance cassette. Flanking of *ermB* with *loxP* sites enabled us to flox out the resistance cassette by the Cre recombinase constitutively produced by the vector pRAB1 [64]. This generated a marker-free mutant minimizing downstream effects caused by the resistance cassette.

### 4.5. Genomic Library

Chromosomal DNA from *S. carnosus* was isolated [57] using lysostaphin (0.5 mg/mL) for cell lysis. Genomic DNA was sheared using the nebulizer from the TOPO Shotgun Subcloning Kit (Invitrogen, Carlsbad, CA, USA) and separated on a preparative agarose gel. Five pools of DNA from 1.7 to 3 kbp were isolated, cleaned and treated with Klenow fragment to generate blunt ends. DNA from each pool was used for ligation into pKT25 [65], which had been cut with *SmaI* and dephosphorylated by rAPid Alkaline Phosphatase (Roche, Mannheim, Germany). The resulting plasmids were transformed into NEB 5-alpha electrocompetent *E. coli* cells (New England Biolabs, Ipswich, MA, USA) according to the manufacturer’s protocol. Ligation procedure was performed twice, resulting in ten transformations. Each transformation was plated on three LB/Kanamycin (50 µg/mL) plates (15 cm in diameter) and incubated overnight at 37 °C. All colonies from the same pool were combined and stored in 50% glycerol at −80 °C. From each pool, an aliquot was used to inoculate LB media for plasmid isolation (“prey” vectors). Over 80% of the plasmids contained genomic DNA inserts, and cell wall related genes like *pbpA*, *pbpF*, *pbp4*, *rodA* and *murJ* could each be amplified in at least one subpool. SCA_1084 (*pbp2*) was cloned into pUT18C [65] as “bait” vector.

### 4.6. Bacterial-Two-Hybrid Assays

The method of Karimova et al. was used [65]. The plasmids were co-transformed in *E. coli* BTH101, plated on LB agar plates containing 100 μg/mL ampicillin, 50 μg/mL kanamycin and 100 μg/mL streptomycin and incubated for 16 h at 37 °C. Ten colonies per each interaction tandem were transferred onto MacConkey agar plates (100 μg/mL ampicillin, 30 μg/mL kanamycin and 100 μg/mL streptomycin) and incubated for 24–48 h at 30 °C. To generate triplicates, the first three colonies per each interaction tandem were inoculated overnight in LB media containing 100 μg/mL ampicillin, 30 μg/mL kanamycin, 100 μg/mL streptomycin and 0.5 mM IPTG. Overnight cultures were diluted in H_2_O_bidest_ (1:4) and cell density was measured at OD_600_. Of each cell suspension, 100 µL was mixed with 20 µL 0.1% SDS, 40 µL chloroform and 1 mL Z buffer (70 mM Na_2_HPO_4_ × 12 H_2_O, 30 mM NaH_2_PO_4_ H_2_O, 1 mM MgSO_4_, 0.2 mM MnSO_4_, pH 7, 100 mM β-mercaptoethanol). The cells were solubilized by pipetting 10–15 times. Of each supernatant, 100 µL was incubated with 20 µL ONPG solution (ortho-Nitrophenyl-β-galactoside; 4 mg/mL ONPG in Z-buffer without β-mercaptoethanol) for 10 min. ONPG served as substrate for the detection of β-galactosidase activity. The colorless substrate is cleaved to galactose and the yellow colored product ortho-nitrophenol. The reaction was stopped by adding 50 µL of 1 M Na_2_CO_3_ and the optical density was determined at 420 nm. The enzymatic activity of the β-galactosidase is given in units/mg dry weight bacteria and was calculated as follows:

Calculation of enzyme activity:

A = 200 × (OD_420_ − OD_420_ in control tube)/minutes of incubation × dilution factor

Calculation of bacteria dry weight:

1 mL of culture at OD_600_ = 1 corresponds to 300 μg dry weight bacteria

A negative control was performed by co-transforming the empty plasmids pKT25 and pUT18C into the host strain *E. coli* BTH101. The cut-off value for positive interactions was four times the negative control.

### 4.7. Peptidoglycan Analysis

The cells were grown in 20 mL BM for 8 h and peptidoglycan was isolated and analyzed by UPLC and UPLC/MS as previously described [37]. Precursors were isolated as published [66] and analyzed by UPLC-MS using the PGN method.

### 4.8. Overproduction of SCA_0214

Main cultures (20 mL B0 media supplemented with either 5 mM glucose for repression or 25 mM xylose for induction) were inoculated with overnight cultures to OD_578_ = 0.05. After 8 h culture, supernatant was used to isolate extracellular proteins using StrataClean beads (Agilent Technologies, St. Clara, CA, USA). Intracellular proteins were extracted from pelleted cells. Cells were disrupted using a FastPrep^®^-24 (MP Biomedicals, Santa Ana, CA, USA) and 500 μL acid washed glass beads (0.22 mm in diameter, Sigma-Aldrich). Cells were treated 4 times at 6500 rpm for 30 s. After two cycles, the samples were placed on ice for 5 min to reduce potential heat that develops during FastPrep^®^-24 treatment and that could lead to protein denaturation. Afterwards, the tubes were centrifuged at 12,000 rpm for 5 min at RT. The supernatant contained the intracellular proteins. Proteins were analyzed by SDS-PAGE stained with Coomassie.

## 5. Conclusions

We could show that the PGN of the apathogenic bacterium *S. carnosus* TM300 differs from the well-studied PGN of the pathogen *S. aureus*. In TM300 free stem peptides are shortened to contain tri peptides only in contrast to the unmodified penta peptides of *S. aureus*. This suggests the activities of an ld- and a dd-CP. We could show, that the proposed dd-CP might be regulated by three enzymes that interact with the PGN synthase PBP2: HlyD, FtsE and FtsX. The overexpression of the respective operon results in the reappearance of penta stem peptides. The ld-CP seems to be regulated by the catabolite control protein A (*ccpA*) as high sugar concentrations result in the appearance of tetra stem peptides. Taken together, our results show that the PGN of *S. carnosus* can adapt to various conditions.

## Figures and Tables

**Table 1:** Muropeptides of *S. carnosus* TM300 analyzed by UPLC-MS. Each muropeptide contains one to five stem-peptides and each consists of the three to five amino acids l-Ala-d-iGln-l-Lys(-d-Ala)(-d-Ala). The interpeptide bridges are built of Gly molecules. The sum of all Gly residues present in each muropeptide is given. Muropeptide peaks 4, 7 and 10 (highlighted in bold) are strongly elevated by *hlyD-ftsE-ftsX* overexpression. Muropeptide peaks 3, 6 and 9 (highlighted in italics) are strongly elevated by high glucose and fructose concentrations.

Peak	Retention Time (min)	M + H^+^ (Da)	Proposed Molecular Formula	Lengths of the Stem Peptides	Inter-Peptide Bridges
					Gly
1	7.5	1111.5192	C_43_H_75_N_12_O_22_^+^	Tri	5
2	8.6	1036.4937	C_40_H_70_N_13_O_19_^+^	Tetra w/o GlcNAc	6
		1093.5164	C_42_H_73_N_14_O_20_^+^		7
		1150.5417	C_44_H_76_N_15_O_21_^+^		8
		1207.5699	C_46_H_79_N_16_O_22_^+^		9
		**1239.5699**	**C_48_H_83_N_14_O_24_^+^**	Tetra	6
		1296.5939	C_50_H_86_N_15_O_25_^+^		7
		1353.6135	C_52_H_89_N_16_O_26_^+^		8
		1410.6370	C_54_H_92_N_17_O_27_^+^		9
*3*	*10.2*	*979.4691*	*C_38_H_67_N_12_O_18_*^+^	*Tetra* w/o *GlcNAc*	*5*
		*1182.5502*	*C_46_H_80_N_13_O_23_*^+^	*Tetra*	*5*
**4**	**11.1**	**1253.5856**	**C_49_H_85_N_14_O_24_^+^**	**Penta**	**5**
5	19.2	2275.0595	C_89_H_152_N_25_O_44_^+^	Tri-Tetra	10
*6*	*21.5*	*2346.0815*	*C_92_H_157_N_26_O_45_*^+^	*Tetra*_2_	*10*
**7**	**21.9**	**2417.1143**	**C_95_H_162_N_27_O_46_^+^**	**Penta-Tetra**	**10**
8	25.4	3438.5927	C_135_H_229_N_38_O_66_^+^	Tri-Tetra_2_	15
*9*	*27.2*	*3509.6465*	*C_138_H_234_N_39_O_67_*^+^	*Tetra*_3_	*15*
**10**	**27.5**	**3580.6553**	**C_141_H_239_N_40_O_68_^+^**	**Penta-Tetra_2_**	**15**
11	29.1	4602.0920	C_181_H_306_N_51_O_88_^+^	Tri-Tetra_3_	20
12	31.6	5765.6142	C_227_H_383_N_64_O_110_^+^	Tri-Tetra_4_	25

Note: w/o—without.

**Table 2:** BTH interaction of proteins related to PGN biosynthesis. Interactions between proteins putatively involved in PGN biosynthesis were tested by BTH experiments. The mean values of three independent experiments are given in Miller Units per mg dry weight bacteria. (Gray: <460; orange: 1500–3000; red: >3000)

	pUT18C					
**pKT25**	**Units/mg**	***pbp2***	***ftsX***	***ftsE***	***hlyD***	***zip***
	***pbp*2**	3769 ± 626	4373 ± 105	136 ± 13	3488 ± 658	164 ± 28
	***fts*X**	3714 ± 833	2502 ± 491	2008 ± 289	2842 ± 287	165 ± 25
	***fts*E**	109 ± 21	4484 ± 279	354 ± 138	132 ± 27	201 ± 103
	***hly*D**	4020 ± 431	2280 ± 456	205 ± 29	3515 ± 113	156 ± 36
	**zip**	113 ± 20	155 ± 4	126 ± 7	157 ± 59	6608 ± 546

**Table 3:** Determination of colony size. The size of ten individual grown colonies was measured digitally. The *ccpA* mutant showed a 57% decrease in cell size that could be complemented again. The influence of glucose was only minor. The mean values of three independent experiments are given.

Strain	5 mM Glucose (mm)	25 mM Glucose (mm)
*S. carnosus* TM300	0.91 ± 0.07	0.83 ± 0.09
*S. carnosus* TM300 Δ*ccpA*	0.39 ± 0.07	0.33 ± 0.06
*S. carnosus* TM300 Δ*ccpA* compl.	0.92 ± 0.04	0.89 ± 0.07

**Table 4:** Calculation of growth rate. Growth rate for each strain was calculated for the mid exponential phase between time point 4 and 5 h and is given as doubling time per hour (t_d_/h). *S. carnosus* TM300 Δ*ccpA* showed a decreased doubling time compared to its parental strain. The influence of high glucose concentrations was only minor. The mean values of three independent experiments are given.

Strain	5 mM Glucose (t_d_/h)	25 mM Glucose (t_d_/h)
*S. carnosus* TM300	1.82 ± 0.16	2.10 ± 0.35
*S. carnosus* TM300 Δ*ccpA*	0.71 ± 0.31	0.67 ± 0.19
*S. carnosus* TM300 Δ*ccpA* compl.	1.90 ± 0.40	2.00 ± 0.38

## Supplementary Materials

The following are available online at www.mdpi.com/2079-6382/5/4/33/s1. Figure S1: Muropeptide structures of *S. aureus* and *S. carnosus* TM300; Figure S2: Growth behavior of *S. carnosus* TM300 Δ*hlyD*-*ftsEX*; Figure S3: Deletion of *hlyD-ftsEX* and overexpression of single genes does not influence the peptidoglycan pattern; Figure S4: Analysis of the *S. carnosus* TM300 Δ*0214::ermB* mutant and localization of the encoded protein; Figure S5: Influence of different sugars on the peptidoglycan of *S. carnosus* TM3; Figure S6: Influence of glucose on the peptidoglycan of *S. aureus* SA113 Δ*spa*; Table S1: Bacterial strains; Table S2: Plasmids; Table S3: Oligonucleotides.

## Abbreviations

The following abbreviations are used in this manuscript:

BTH	Bacterial-Two-Hybrid
PGN	peptidoglycan
UPLC/MS	Ultra Performance Liquid Chromatography coupled to mass spectrometry
MurNAc	*N*-acetylmuramic acid
GlcNAc	*N*-acetylglucosamine
CP	carboxypeptidase
