# Supplementary material for: The Peptidoglycan Pattern of Staphylococcus carnosus TM300—Detailed Analysis and Variations Due to Genetic and Metabolic Influences
Source: Antibiotics (Basel). 2016 Sep 23;5(4):33. doi: 10.3390/antibiotics5040033 (PMC5187514; doi:10.3390/antibiotics5040033)
Supplement: Supplementary file 1 [file antibiotics-05-00033-s001.pdf]

Supplementary Materials: The Peptidoglycan Pattern of *Staphylococcus carnosus* TM300—Detailed Analysis and Variations Due to Genetic and Metabolic Influences

Julia Deibert, Daniel Kühner, Mark Stahl, Elif Koeksoy and Ute Bertsche

Table S1. Bacterial strains.

Bacterial Strain	Relevant Genotype	Source
Escherichia coli BTH101	Bacterial-Two-Hybrid strain F⁻, *cya-99 araD139 galE15 galK16 rpsL1 (Str^r) hsdR2 mcrA1 mcrB1*	(Karimova *et al.*, 2005)
Escherichia coli NEB 5-alpha	Electrocompetent cloning strain *fhuA2Δ(argF-lacZ)U169 phoA glnV44 Φ80Δ (lacZ)M15 gyrA96 recA1 relA1 endA1 thi-1 hsdR17*	New England Biolabs GmbH
Escherichia coli XL1-Blue	Cloning strain *recA1 endA1 gyrA96 thi-1 hsdR17(rK⁻, mK⁺) supE44 relA1 lac [F′ proAB lacIqZΔM15::Tn10(tetr)]*	Promega GmbH
Escherichia coli DH5α	*General cloning host ((φ 80d lacZΔM15) Δ(lacZYA-argF) recA endA1 hsdR17 supE44 thi-1 gyrA96 relA1 deoR)*	(Hanahan, 1983)
Escherichia coli DC10B	*dam+Δdcm ΔhsdRMS endA1 recA1*	(Monk *et al.*, 2012)
Staphylococcus carnosus TM300	Wild type (SK311)	(Schleifer & Fischer, 1982), (Götz, 1990)
Staphylococcus aureus N315	Methicillin resistant strain isolated in 1982	(Kuroda *et al.*, 2001)
Staphylococcus aureus SA113 *Δspa*	*rsbU tcaR agr* mutant and three prophages, Φ 11, −12, and −13 protein A deletion mutant (*spa*)	(Herbert *et al.*, 2010)
Staphylococcus aureus RN4220	Derivative of *S. aureus* NCTC 8325-4, acceptor for foreign DNA	(Iordanescu & Surdeanu, 1976)

Table S2. Plasmids.

Plasmid	Relevant Marker	Source
pJET	High efficiency vector system for positive selection of PCR products	Thermo Fisher Scientific
pKT25	Derivative of low copy-number pSU40, carrying the first 224 amino acids of *B. subtilis* CyaA (T25 fragment), upstream of a multiple cloning site; Kan^R	(Karimova *et al*., 2005)
p25N	Derivative of low copy-number pSU40, carrying gene encoding the first 224 amino acids of CyaA (T25 fragment), downstream of a multiple cloning site; Kan^R	(Claessen *et al*., 2008)
pKT25-zip	Derivative of low copy-number pSU40, carrying the first 224 amino acids of *B. subtilis* CyaA (T25 fragment), upstream of a multiple cloning site; 35-aa-long leucine zipper derived from protein GCN4, Kan^R	(Karimova *et al*., 2005)
pKT25-genomic library fragment	Genomic library of *S. carnosus* TM300 harboring gene fragments of 1000–3000 bp to cover up most of the genes involved in cell wall biosynthesis	This work
pUT18C	Derivative of high copy-number pUC19, carrying gene encoding amino acids 225 to 399 of CyaA (T18 fragment), upstream of a multiple cloning site; Amp^R	(Karimova *et al*., 2005)
pUT18	Derivative of high copy-number pUC19, carrying gene encoding amino acids 225 to 399 of CyaA (T18 fragment), downstream of a multiple cloning site; Amp^R	(Karimova *et al*., 2001)
pUT18C-zip	Derivative of high copy-number pUC19, carrying gene encoding amino acids 225 to 399 of CyaA (T18 fragment), upstream of a multiple cloning site; 35-aa-long leucine zipper derived from protein GCN4, Amp^R	(Karimova *et al*., 2001)
pUT18C-Sca_1084	pUT18C containing T18 fused in frame to the 5′ end of *S. carnosus pbp2*; Amp^R	This work
pUT18C-Sca_1995	pUT18C containing T18 fused in frame to the 5′ end of *S. carnosus* hypothetical protein Sca_1995; Amp^R	This work
pUT18C-Sca_1996	pUT18C containing T18 fused in frame to the 5′ end of *S. carnosus* hypothetical protein Sca_1996; Amp^R	This work
pUT18C-Sca_1997	pUT18C containing T18 fused in frame to the 5′ end of *S. carnosus* hypothetical protein Sca_1997; Amp^R	This work
pKT25-Sca_1084	pKT25 containing T25 fused in frame to the 5′ end of *S. carnosus pbp2*; Kan^R	This work
pKT25-Sca_1995	pKT25 containing T25 fused in frame to the 5′ end of *S. carnosus* hypothetical protein Sca_1995; Kan^R	This work
pKT25-Sca_1996	pKT25 containing T25 fused in frame to the 5′ end of *S. carnosus* hypothetical protein Sca_1996; Kan^R	This work
pKT25-Sca_1997	pKT25 containing T25 fused in frame to the 5′ end of *S. carnosus* hypothetical protein Sca_1996; Kan^R	This work

Table S3. Oligonucleotides.

Primer	Sequence	Restriction Enzyme	Construct Description
pUT18C for	AGCGGACGTTCGAAGTTCTC		Sequencing
pUT18C rev	GGAGCAGACAAGCCCGTCAGG		
pUT18 rev	CTCGGTGCCCACTGCGGAAC		
pKT25 for	ATTATGCCGCATCTGTCC		
pKT25 rev	TGCTGCAAGGCGATTAAG		
Sca_1084 for	TATATAGGATCCGCGTATGACGGAA AG	*BamHI*	pUT18-Sca-*pbp2* and pKT25-Sca-*pbp2*
Sca_1084 rev	TATATAGAGCTCTATAAAACGCGAC AAGC TC	*SacI*	
Sca_1995 for	TATATAGGATCCAAGAGGTGGTACG ATGA AT	*BamHI*	pUT18-Sca_1995 and pKT25-Sca_1995
Sca_1995 rev	TATATAGGTACCTCCCAACTTCCTTT ATTT GA	*KpnI*	
Sca_1996 for	TATATAGGATCCAATGATTAAATTA AAGC ATGTC	*BamHI*	pUT18-Sca_1996 and pKT25-Sca_1996
Sca_1996 rev	TATATAGGTACCATTCATCGTACCA CCTCT TC	*KpnI*	
Sca_1997 for	TATATAGGATCCCTTGAAGAAGAAA TTGA TTTG	*BamHI*	pUT18-Sca_1997 and pKT25-Sca_1997
Sca_1997 rev	TATATAGGTACCTGACATGCTTTAA TTTA ATC	*KpnI*	
Sca_1995–1997 up for	GGTACCATATCAATTCGGCTGTATC	*KpnI*	Sca_1995–1997 knock out construct upstream fragment
Sca_1995–1997 up rev	atgtcccaaCCCGGGCTCCTATCCATATT ATT C	*SmaI*	Sca_1995–1997 knock out construct upstream region; overlap region to downstream fragment
Sca_1995–1997 down for	ggataggagCCCGGGTTGGGACATGTTG AAT AC	*SmaI*	Sca_1995–1997 knockout construct downstream region; overlap region to upstream fragment
Sca_1995–1997 down rev	GTCGACCCCTTCCCTTAATTTAATTG	*SalI*	Sca_1995–1997 knockout construct; downstream fragment
ermB-for	CCCGGGTACCGTTCGTATAGCATAC A	*SmaI*	*ermB* cassette binding at l*ox71*
ermB-rev	CCCGGGTACCGTTCGTATAATGTAT G	*SmaI*	*ermB* cassette binding at l*ox66*
Sca_1997 for-TTG	GGATCCTAAATT**AGGAGG**TATTAAT T*TTG*A AGAAGAAATTGATTTGG	*BamHI*	native start codon TTG, full operon Sca_1995–1997
Sca_1997 for ATG	GGATCCTAAATT**AGGAGG**TATTAAT T*ATG*A AGAAGAAATTGATTTGGA	*BamHI*	optimized start codon ATG; full operon Sca_1995–1997
Sca_1996 for	GGATCCTAAATT**AGGAGG**TATTAAT TATGA TTAAATTAAAGCATGTC	*BamHI*	truncated operon Sca_1995–1996
Sca_1995 rev	CCCGGGTTATTTGATAATATCAATC AATTC	*SmaI*	full and truncated operon
Sca_1995–1997 for pRAB11-EF-Tu	ttaaatattatttttaattagaacttactaacaaacaagga ggaaagaacaTTGAAGAAGAAATTGATT TGGATA ATTTC		Complementation for cloning into pRAB11-EF-Tu
Sca_1995–1997 rev pRAB11-EF-Tu	aaacgacggccagtgttaTTATTTGATAATAT CAA TCAATTCTTTTTTAG		
Sca_0214 KO Down-for	tcgagctcggtacccTAAAGATGGACCGTT TGC		overlapping region to pGS1

Table S3. *Cont.*

Primer	Sequence	Restriction Enzyme	Construct Description
Sca_0214 KO Down-rev	gtttcttttgcccgggCGTTTATTATTCCTCC TAAC TATG	*SmaI*	overlapping region to upstream fragment; additional *SmaI* restriction site
Sca_0214 KO Up-for	ataataaacgcccgggCAAAAGAAACATTA ATAT GACCG	*SmaI*	overlapping region to downstream fragment; additional *SmaI* restriction site
Sca_0214 KO Up-rev	ctctagaggatccccAGCGATAACAGTCTT ACG		overlapping region to pGS1
Sca_0214 SD *BamHI* for	GGATCCTAAATT**AGGAGG**TATTAAT TATGA AGAATTTGATTAAAC	*BamHI*	Complementation and overexpression construct for pPTX
Sca_0214 *SmaI* rev	CCCGGGTTATGAACATCCACTCTC	*SmaI*	
KO Sca_0214 flanking for	TGGCGCACTAGGCCAAATC		KO confirmation primer
KO Sca_0214 flanking rev	ACCGCAGCAGTACCTGTTC		
Up SAOUHSC01850 for	tagaattcgagctcccATTCCAAATTGGTGC ACG		Up overlap Down
Up SAOUHSC01850 rev	ttttgtgaatAATTTCCTCCTTGTAAACGT TTTAT TC		Up overlap pMAD
Down SAOUHSC01850 for	gaggaaattATTCACAAAATTAGGCATT CATC		Down overlap pMAD
Down SAOUHSC01850 rev	catgccatggtacccCAGATAAGTTATTAC AATAT CGATTTC		Down overlap Up
SAOUHSC01850 for *BglII*	agatctcttactaacaaacaaggaggaaagaacaAT GA CAGTTACTATATATGATGTAG	*BglII*	Complementation for cloning into pRAB11-EF-Tu
SAOUHSC01850 rev *EcoRI*	gaattcttaTTATTTTGTAGTTCCTCGGTA TTC	*EcoRI*	
SAOUHSC01850 flanking KO for	TTAAACCACGTACATCAC		KO confirmation primer
SAOUHSC01850 flanking KO rev	CATTAGAACAGCAACAAG		

Notes: Restriction sites used for cloning procedure are underlined. Overlapping regions are shown in small form letters. Shine-Dalgarno sequence is highlighted in bold letters.

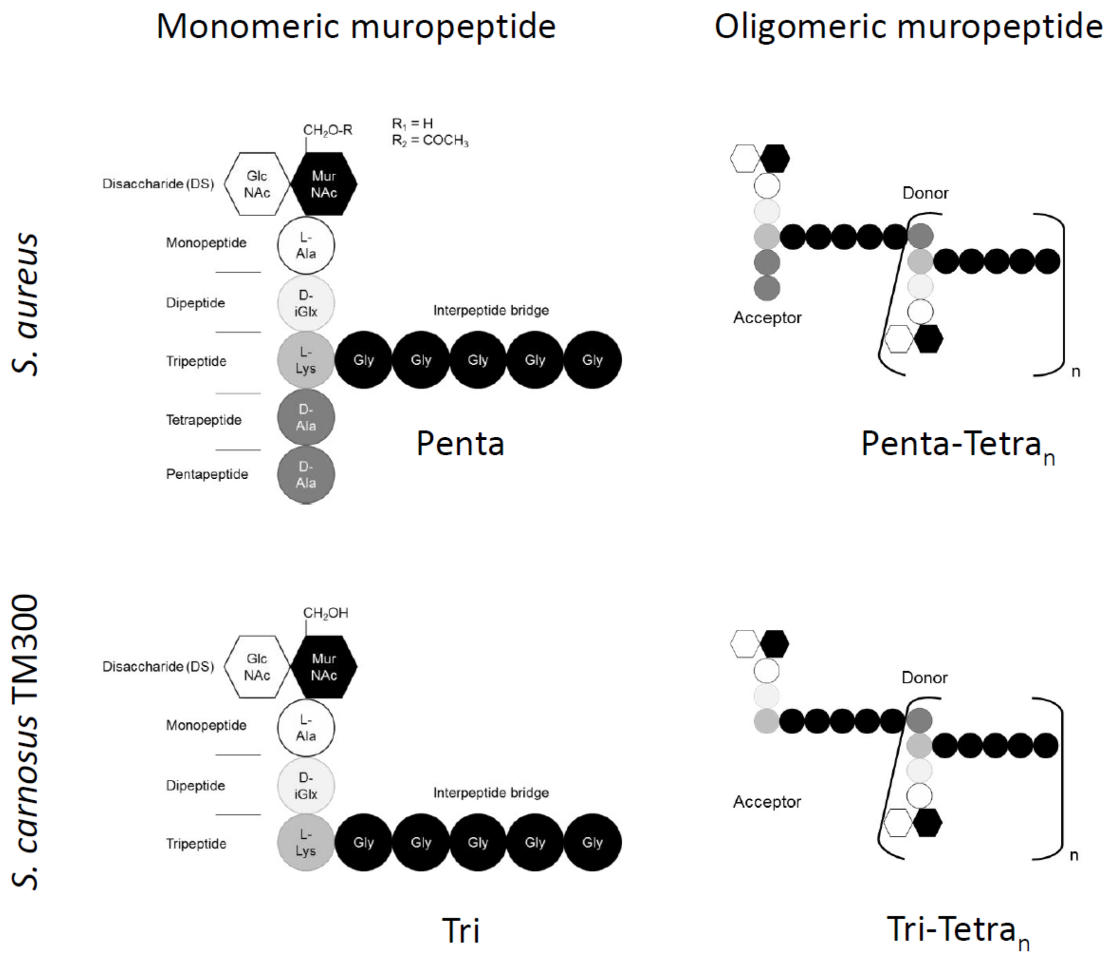

Figure S1. Muropeptide structures of *S. aureus* and *S. carnosus* TM300. The PGN can be isolated and enzymatically digested into monomeric or oligomeric muropeptides. Monomeric muropeptides contain a disaccharide moiety (β-1,4-linked GlcNAc-MurNAc) and stem peptide, which in the case of *S. aureus* consists of L-Ala – DiGlu – L-Lys – D-ALA – D-Ala. Most of the D-iGlu had been amidated to D-iGln. Both are referred to as D-iGlx. The stem peptides can be cross-linked resulting in oligomeric muropeptides. The necessary transpeptidation reaction is performed between D-Ala on position four of the donor stem peptide and the N-terminal Gly of the interpeptide bridge of the acceptor stem peptide by the expense of the last D-Ala of the donor stem peptide. The resulting tetra stem peptide of the original donor can serve as an acceptor for further cross-linking reactions. In *S. carnosus* TM300 the penta stem peptide of the first acceptor or of free acceptors is shortened to a tripeptide. GlcNAc: N-Acetylglucosamine; MurNAc: N-Acetylmuramic acid.

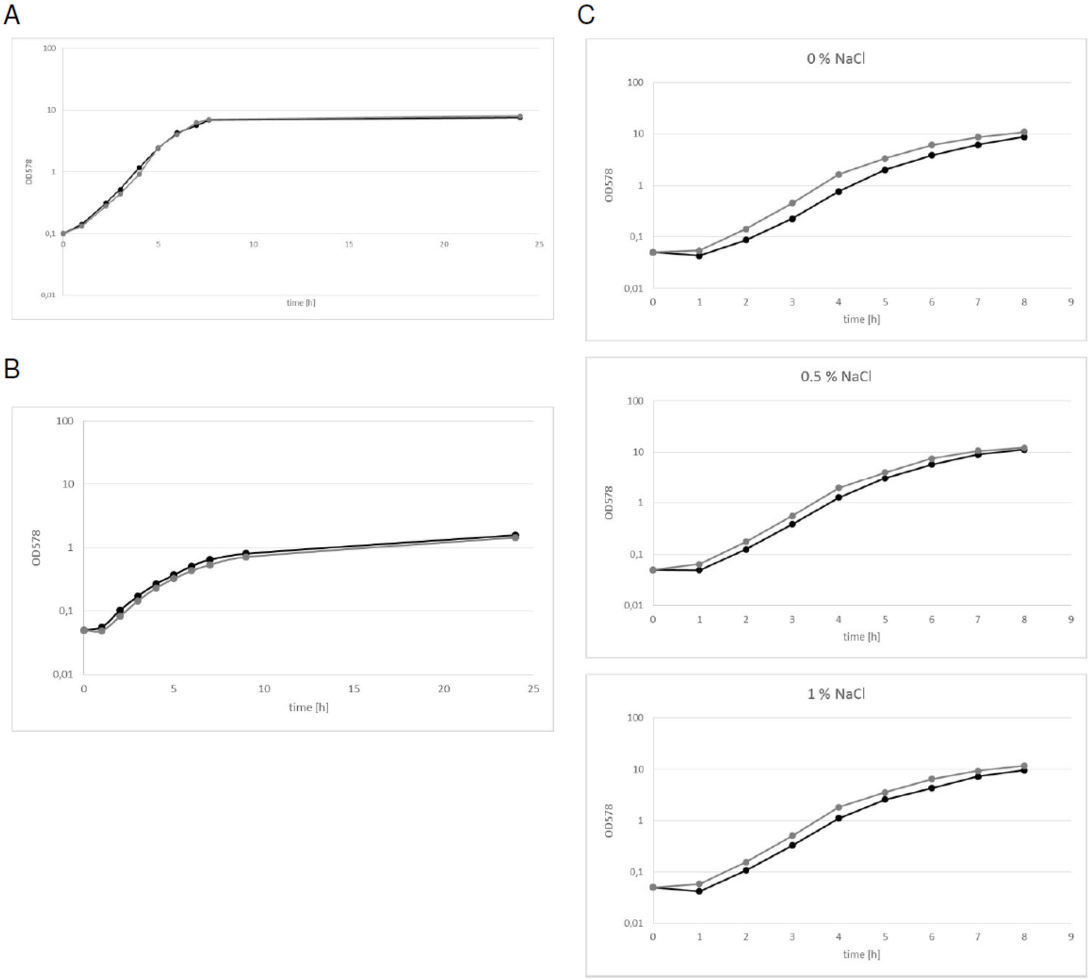

Figure S2. Growth behavior of *S. carnosus* TM300 Δ*hlyD-ftsEX*. (**A**) The *S. c. ΔhlyD-ftsEX* strain grown in B media showed no difference in growth behavior under standard conditions compared to *S. c.* TM300. (**B**) Limitation of nutrients has no influence on the growth behavior of *S. c.* TM300 *ΔhlyD-ftsEX* compared to the wild type strain *S. c.* TM300. Minimal media was supplemented with 25 mM glucose. (**C**) Different NaCl concentrations did not influence the growth behavior of *S. c.* TM300 *ΔhlyD-ftsEX* compared to the wild type. Growth curves were performed with B media containing either 0%, 0.5% or 1% NaCl. Black circles: *S. carnosus* TM300; Grey circles: *S. carnosus* TM300 Δ*hlyD-ftsEX*.

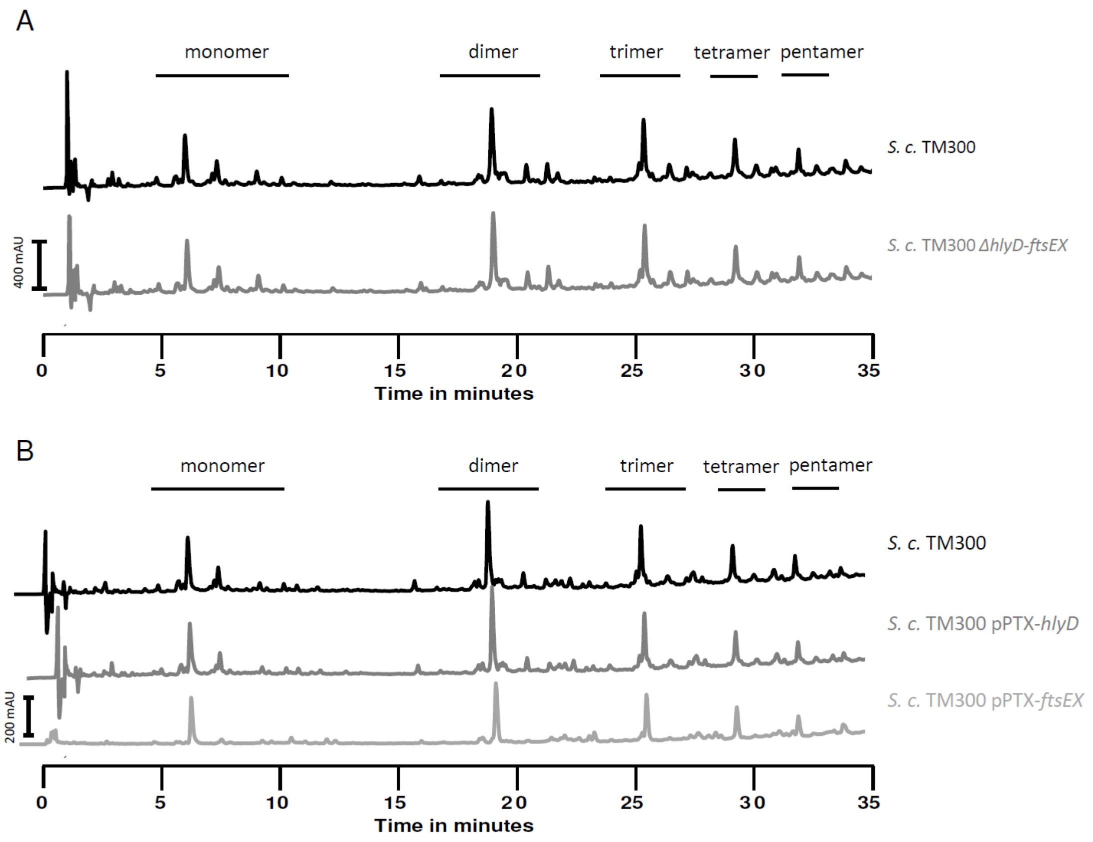

Figure S3. Deletion of *hlyD-ftsEX* and overexpression of single genes does not influence the peptidoglycan pattern. Main cultures were grown in B media with 25 mM xylose and cells were harvested after 8 h of growth. PGN was isolated and analyzed by UPLC. (**A**)No difference between the wild type strain and the *hlyD-ftsEX* deletion mutant could be detected. (**B**) PGN is not altered when either *hlyD* or *ftsEX* alone are overexpressed in *S. c.* TM300.

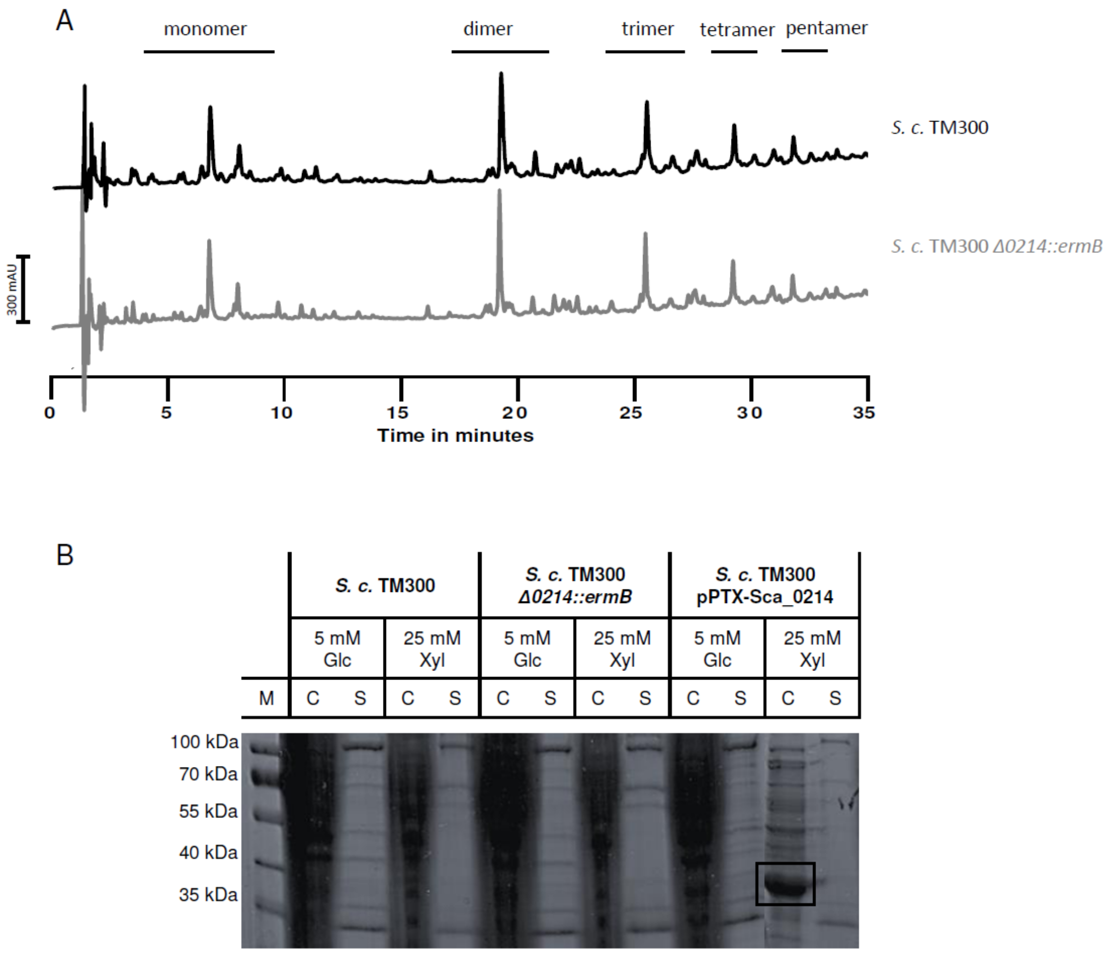

Figure S4. Analysis of the *S. carnosus* TM300 *Δ0214::ermB* mutant and localization of the encoded protein. (**A**) Main cultures were grown for 8 hours in BM supplemented with 5 mM glucose. Deletion of Sca_0214 had no effect on the PGN pattern. (**B**) The putative LD-CP (Sca_0214) was overproduced in *S. carnosus* TM300 to investigate its localization. Protein production in *S .c.* TM300 pPTX-Sca_0214 was induced by 25 mM xylose (Xyl) or repressed by 5 mM glucose (Glc). Cells were harvested after 8 hours of growth and cytoplasmic (C) and extracellular proteins of the supernatant (S) were isolated and analyzed by SDS-PAGE. The LD-CP was localized in the cytoplasm (box). The calculated mass of the LD-CP is 39 kDa. In addition, the extracellular (S) protein fractions did not differ in the wild type, the *S. c.* TM300 *Δ0214::ermB* mutant and the Sca_0214 overproduction strain, indicating that Sca_0214 has no influence on protein secretion.

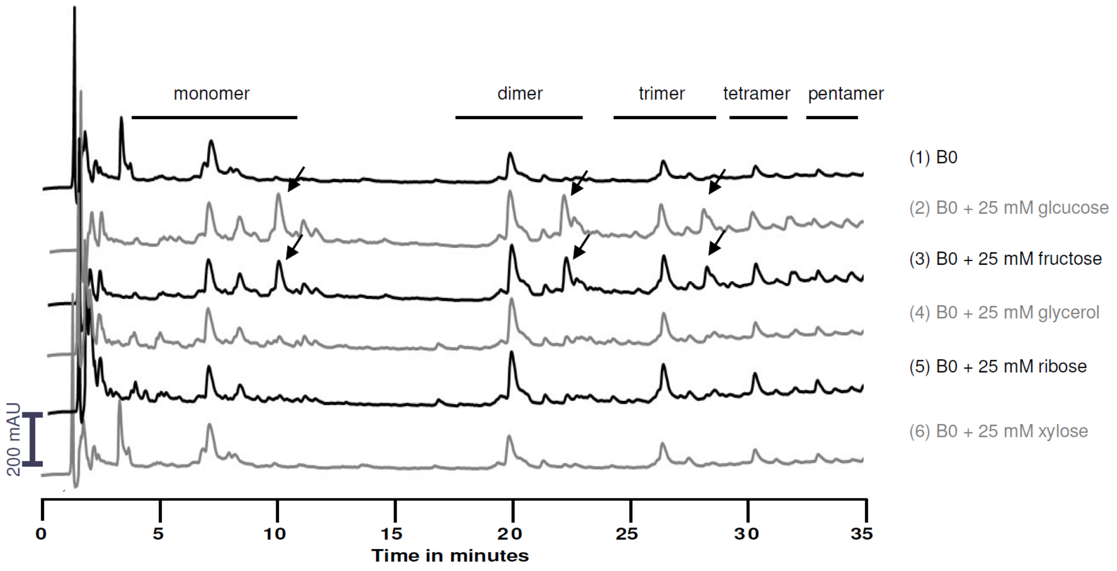

Figure S5. Influence of different sugars on the peptidoglycan of *S. carnosus* TM300. Main cultures were grown for 8 h in B0 media supplemented with either 25 mM glucose (panel 2), fructose (panel 3), glycerol (panel 4), ribose (panel 5), or xylose (panel 6), or without an additional carbon source (panel 1). Peptidoglycan analysis showed clearly enlarged peaks in the monomer to trimer fractions when cells had been grown in the presence of 25 mM glucose or fructose (arrows in panels 2 and 3). Glycerol, ribose and xylose did not lead to an altered peptidoglycan pattern, nor were the cells affected when no additional carbon was added to the medium (panel 1).

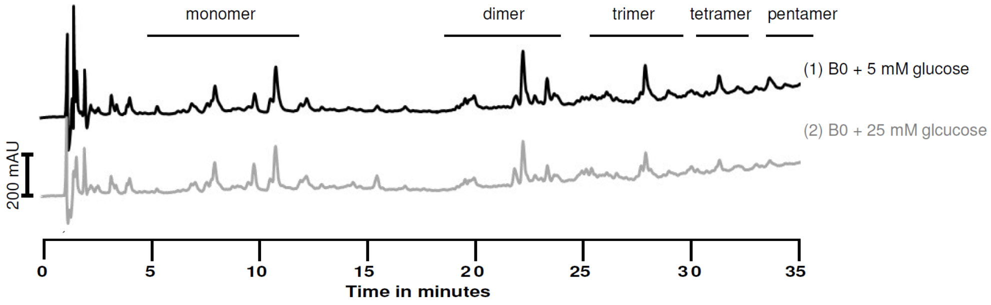

Figure S6. Influence of glucose on the peptidoglycan of *S. aureus* SA113 *Δspa.* Main cultures were grown for 8 h in B0 media supplemented with either 5 or 25 Mm glucose. PGN was isolated and analyzed by UPLC. The muropeptide pattern of *S. aureus* SA113 *Δspa* showed no difference when grown in low (panel 1) or high (panel 2) glucose concentrations.

© 2016 by the authors; licensee MDPI, Basel, Switzerland. This article is an open access article distributed under the terms and conditions of the Creative Commons by Attribution (CC-BY) license (http://creativecommons.org/licenses/by/4.0/).
